# No speed limit now for publication of research in Mediators of Inflammation

**DOI:** 10.1155/S0962935192000012

**Published:** 1992

**Authors:** Iván L Bonta


### Editorial

Mediators of Inflammation 1, 3–4 (1992)

### No speed limit now for publication of research in *M*ediators of *I*nflammation

While research on inflammation processes continues to be an expanding branch within the life sciences there has been a spectacular growth within the field of mediators. Mediators constitute the fundamental network of signal and effector molecules of communication between cells participating in the events of inflammation. We anticipate that the bidirectional communication between the immune and the nervous system will also prove to involve mediators. Furthermore these molecules represent important targets for the development of drugs which could be applied in the treatment of inflammatory disorders. Accordingly, *Mediators of Inflammation* is of interest to those researchers involved in cell biology, immunology, pharmacology, clinical sciences and the pharmaceutical industry.

While not neglecting any of the earlier known mediators (such as histamine, bradykinin, prostaglandins, leukotriences and PAF), *Mediators of Inflammation* welcomes original articles on cytokines (tumour necrosis factors, interleukins, interferons), evasive molecules e.g., nitric oxide, the family of cell adhesion promoting molecules and the biological response modifiers. Priority of publication will be given to articles in which there is emphasis on interactions between various classes of mediators. Articles dealing with pharmacological influences on mediators of inflammation will be particularly welcome.

From the viewpoint of pathophysiology of inflammatory disorders there is an intriguing question: should mediators be counteracted or, by contrast, can they be utilised as therapeutic agents? We faced a similar problem 10 years ago in that in immunoinflammatory conditions prostaglandin $E_2$ exerts a two-fold function, i.e., reinforcement of the vascular inflammatory components and inhibitory modulation of macrophage-mediated immunological events. This functional dualism of prostaglandin $E_2$ was reminiscent of Janus, the ancient Roman diety, traditionally depicted with two faces. Occasionally artists have shown the faces in a way which reflects both hostility and benevolence. The analogy with prostaglandin $E_2$ appeared strong, because this mediator was shown to exert both harmful and beneficial functions. While some aspects of this concept appeared speculative, not only is the evidence in favour of it now firm, but recent findings indicate that the essentials of simultaneously being a major culprit and a physiologically beneficial regulator can be extended to other mediators of inflammation. Examples are represented by the efforts of counteracting interleukin-1 or tumour necrosis factor in some clinical conditions and by recent trials in harnessing their beneficial functions in other disorders. The functional dualism is likely to be valid for several mediators of inflammation. This, in fact prompted us to chose Janus the *par excellence* symbol of dualism as the logo for *Mediators of Inflammation.* Because dualisms is by definition controversial we welcome articles which, while being based on sound experiments, will provoke counteropinions from colleagues.

Introduction and development of new techniques have opened up unexplored areas in mediator research, making it possible to study molecular biological processes and their clinical impact which were until not beyond experimental approach. However the imbalance between the application of ultramodern high-speed technology in research and lack of application of such technology in publishing these achievements, hampers the communication between workers within the fields. *Mediators of Inflammation* sets out to surmount the imbalance between speed of research and delay in appearance of the article. Reduction in publication time will be achieved by exploitation of modern (i.e. electronic) communication techniques between authors, editors (peer reviewers) and the Publisher.

While speed of publication will be a prime feature of *Mediators of Inflammation,* the highest standards (i.e. novelty and reliability of results) will also be ensured, through rigorous refereeing by internationally renowned authorities within the field. With learned journals reviewing is, often very slow, because referees usually do this work in their spare time. *Mediators of Inflammation* rejects this practice. The members of the team of the Editorial Board, all eminent scientists in their field, have been appointed having obtained their consent to be ready to return evaluation of papers within a fortnight. Hence authors will be assured of rapid and high-quality reviewing.

*Mediators of Inflammation* aims to publish papers

© 1992 Rapid Communications of Oxford Ltd.

within 3 months of acceptance. Even exploiting the above described principles, this short publication time cannot be achieved without the co-operation of the authors who are required to follow precisely the Instructions to Authors which appears elsewhere in this issue. Furthermore with papers requiring revision, the manuscript will need to be returned within a week.

*Mediators of Inflammation* feels challenged to meet these goals. By achieving them it aims to shortly become the rapid forum of prime choice within the field. Also on behalf of my colleague B. Boris Vargaftig, we urge you and your co-workers to contribute the latest and best of your results and thus back our new venture.

*Iván L Bonta*
*Editor-in-Chief*

